# A mathematical model for mapping the insecticide resistance trend in the *Anopheles gambiae* mosquito population under climate variability in Africa

**DOI:** 10.1038/s41598-024-60555-z

**Published:** 2024-04-29

**Authors:** Komi Mensah Agboka, Mark Wamalwa, James Mutuku Mutunga, Henri E. Z. Tonnang

**Affiliations:** 1 International Centre of Insect Physiology and Ecology (Icipe), P.O. Box 30772 00100, Nairobi, Kenya; 2 School of Engineering Design and Innovation Pennsylvania State University, University Park, PA 16802 USA; 3 School of Agricultural, Earth, and Environmental Sciences, University of KwaZulu-Natal, Pietermaritzburg, 3209 South Africa

**Keywords:** Susceptible-infected-resistant, Insecticide resistant malaria vector, Spatial modelling, Decision making, Ecology, Mathematics and computing

## Abstract

The control of arthropod disease vectors using chemical insecticides is vital in combating malaria, however the increasing insecticide resistance (IR) poses a challenge. Furthermore, climate variability affects mosquito population dynamics and subsequently IR propagation. We present a mathematical model to decipher the relationship between IR in *Anopheles gambiae* populations and climate variability. By adapting the susceptible-infected-resistant (SIR) framework and integrating temperature and rainfall data, our model examines the connection between mosquito dynamics, IR, and climate. Model validation using field data achieved 92% accuracy, and the sensitivity of model parameters on the transmission potential of IR was elucidated (e.g. *μ*PRCC = 0.85958, *p*-value < 0.001). In this study, the integration of high-resolution covariates with the SIR model had a significant impact on the spatial and temporal variation of IR among mosquito populations across Africa. Importantly, we demonstrated a clear association between climatic variability and increased IR (width = [0–3.78], α = 0.05). Regions with high IR variability, such as western Africa, also had high malaria incidences thereby corroborating the World Health Organization Malaria Report 2021. More importantly, this study seeks to bolster global malaria combat strategies by highlighting potential IR ‘hotspots’ for targeted intervention by National malria control programmes.

## Introduction

Malaria is a life-threatening vector-borne disease caused by parasites (*Plasmodium falciparum*) that are transmitted to humans through the bites of infected female Anopheles mosquitoes. In Africa, the main malaria vectors are *Anopheles gambiae*, *Anopheles coluzzii*, and *Anopheles arabiensis*^1^. These mosquito species are commonly referred to as the *Anopheles gambiae* complex. Over the years, targeting mosquito vectors through the use of insecticides has been critical to the success of national malaria control programs. However, malaria vectors have developed resistance to commonly used insecticides^2,3^. Mosquito resistance mechanisms can be broadly categorized into molecular based target-site mutations, metabolic resistance due to over expression of detoxifying enzymes, behavioural resistance that is occasioned by change in vector activity, and phenotypic resistance attributed to cuticular thickening that slows penetration or sequestration of the insecticides^2,4–7^. The metabolic resistance mechanism is achieved through the overexpression or alteration of detoxifying enzymes that can metabolize the insecticides^8^. The major enzyme families involved in metabolic resistance are Cytochrome P450 monooxygenases, hydrolases, esterases and Glutathione S-transferases^9^. On the other hand, target site resistance is a molecular mechanism that involves mutations in the specific binding site of an insecticide resulting in reduction of the insecticide's binding affinity to its target site in the insect. The two main target site resistance mechanisms are nicotinic acetylcholine receptors (nAChRs) and knock-down (*kdr*) resistance, associated with carbamates/organophosphates and pyrethroids/DDT, respectively^10^.

Previous studies attribute the evolution and wide spread propagation of resistance to recurrent evolution^11^, recombination^12^, and natural selection at specific insecticide resistance loci^13–15^. In addition, vector control programs may contribute to vector population contractions^16^, and reshape genome-wide patterns of diversity. The rapid propagation of insecticide resistance among mosquito populations threatens the effectiveness of current vector control strategies, such as insecticide-treated nets and indoor residual spraying^17^. While there is evidence suggesting an increase in pyrethroid resistance among African malaria vector populations^9,18^, the extensive field studies available do not offer a spatially comprehensive time series of resistance patterns^6^. Moreover, climate variability, with its impact on mosquito population and parasite transmission dynamics, adds another layer of complexity to understand and manage the propagation of resistance^19,20^.

Various strategies have been proposed to circumvent the propagation of insecticide resistance among mosquito vector populations such as the use of bio-pesticides (fungal pathogens)^21^ and biological control agents (*Microsporidia MB*)^22^. However, they are associated with certain limitations such as contamination of the environment and low efficacy in the control of vectors^21,22^. While the WHO has developed a manual with guidelines for insecticide resistance management at the operational vector control level^23^, the adoption of these strategies at the country level is affected by the limited arsenal of available alternative chemistries needed for mosaics, rotations or mixtures. More so, the currently used phenotypic assays for assessing insecticide resistance (technical resistance) do not accurately inform appropriate control of resistant population in the field (practical resistance) due to factors such as environmental conditions and exposure dose^24^. Consequently, there is an urgent need for a comprehensive approach that can accurately predict the spatial patterns of insecticide resistance in the field under various climate scenarios to inform vector control operations.

While studies have reported spatial variation in insecticide resistance within countries^25,26^, or analyzed the spatial trends behind variations across multiple countries, estimating these trends using a data-driven approach is still challenging due to limited available observations of resistant phenotypes in field mosquito populations^2,3^. Furthermore, standardized susceptibility tests for the prevalence of phenotypic resistance in field populations cover a broad geographic range and extend over several decades^6,27^. In addition, the spatial coverage of these data is sparse and uneven, and resistance has rarely been monitored consistently over time, resulting in very few available time series^28^. Additionally, data-driven malaria resistance studies often focus on specific types of resistance due to the nature and quality of available data collected for the particular resistance mechanism^9,18^.

Accurate modeling of the emergence and propagation of insecticide resistance is crucial for the development of effective and sustainable control measures. Furthermore, quantifying the patterns of insecticide resistance will enhance our knowledge of historical resistance propagation in vector populations and aid in the development and optimization of insecticide resistance management strategies^28^. Comprehensive spatial analyses of resistance are also essential for incorporating resistance data into epidemiological models of malaria that inform decision-making at national and global levels^28^. Mathematical models, can be useful tools in providing a general overview of insecticide resistance trends in mosquito populations and they can also be adapted to target specific resistance mechanisms^29–31^. The Susceptible-Infected-Recovered (SIR) model is a widely-used mathematical model for studying disease transmission dynamics^32,33^. Previous studies applied the standard SIR model to analyze malaria transmission through the use and application of the basic reproduction number (${R}_{0}$) to understand malaria transmission patterns in space and time^34^. In this approach, Moukam et al.^34^ simulated the temperature dependence aspect of ${R}_{0}$ to derive the spatial distribution maps for malaria endemicity under different climatic and intervention scenarios. If ${R}_{0}$ < 1 malaria prevalence dies out, while if ${R}_{0}$ > 1 malaria becomes endemic. Incorporating critical environmental factors (that may influence the propagation of resistance mechanisms) in the SIR model can elucidate the emergence and propagation of insecticide resistance mechanisms.

The SIR model offers several advantages over statistical models for modeling trends in space and time. Its’ simplicity and flexibility^35,36^; and mechanistic understanding which facilitates spatial analysis^37,38^ makes it a valuable tool for investigating insecticide resistance transmission dynamics and guiding control strategies. In this study, we present a SIR mathematical model to investigate the spatial trend of the number of individuals within the mosquito population with insecticide resistance in *An. gambiae* populations under the influence of climate variability at a regional scale. Our model combines a SIR framework with environmental factors, such as temperature and rainfall, to capture the intricate relationships between mosquito population dynamics, insecticide resistance, and climate conditions. By integrating high-resolution climate data, we are able to better understand the role of climate variability in shaping the spatial trend of insecticide resistance among *An. gambiae* populations.

The objectives of this study are twofold: (1) to develop a generic mathematical model that approximates the complex interactions between mosquito population dynamics, insecticide resistance, and climate variability; (2) to validate our model using empirical data on *An. gambiae* insecticide resistance ground truthing from multiple sites across a diverse range of climate conditions; that can assist decision-makers to provide policy-relevant insights and recommendations for more effective vector control strategies under various climate scenarios.

While the basic reproduction number (${R}_{0}$) has been extensively used in epidemiology to represent the transmissibility of a disease, in this study, we quantified the evolution of IR using a time-varying population ${R}_{0}$ for female mosquitoes, ignoring differences in genotypes. We parameterised the model using the results of laboratory studies of non-linear temperature and precipitation responses and incorporate these into estimates of the ${R}_{0}$ of insecticide resistance through time and space (Table 1). Consequently, if ${R}_{0}$ < 1, the mosquitoes are suscpetible and no insecticide resistance transmission will occur and therefore, the implemented intervention was successful.

**Table 1:** Model parameters considered in this study.

Parameter (symbol)	Descriptions	Value	Source
${B}_{E}$	Number of eggs per oviposition	200	^57^
${\theta }_{E}$	Daily survival probability of eggs	0.9	^57^
${\theta }_{L}$	Daily survival probability of larvae	0.25	^57^
${\theta }_{p}$	Daily survival probability of pupae	0.75	^57^
${R}_{L}$	Rainfall limit	50 mm	^57^
${k}_{1}$	Constant	0.00554 (◦Cdays) − 1	^57^
${k}_{2}$	Constant	− 0.06737(days) − 1	^57^
$a$	Constant	− 0.3(◦C2days) − 1	^57^
$b$	Constant	1.31(◦Cdays) − 1	^57^
$c$	Constant	− 4.4(days) − 1	^57^
$\alpha$	Offspring not inheriting the resistance rate becomes susceptible again	0.01	Calibrated^1^
$\beta$	The probability that a mosquito becomes resistant due to contact with an insecticide	1	Calibrated^1^
$\gamma$	The probability that a resistant mosquito produces non-resistant offspring	0.75	Calibrated^1^
$\mu i$	Represent the insecticides-induced mortality rate	0.01	Calibrated^1^
$N$	Total population	50	Calibrated^1^

^1^Calibrated parameters were determined through an iterative calibration process, adjusting within realistic ranges until the model’s outputs closely matched observed data on insecticide resistance.

## Results

### Sensitivity analysis and performance assessment

**Figure 1:**
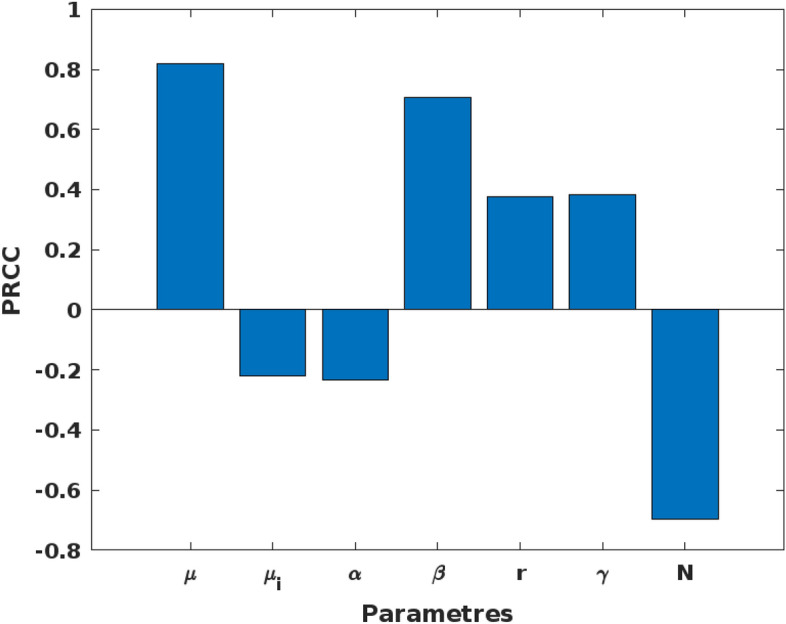
A tornado plot of partial rank correlation coefficient (PRCC) between various variables used in the model. The sensitivity analysis shows changes in the natural birth rate of mosquitoes ($r$), the natural rate of resistance ($\gamma$), the transmission rate ($\beta$), the natural death rate of mosquitoes ($\mu$), the insecticide-induced mortality rate ($\mu i$), and the mortality rate due to offspring of resistant mosquitoes that do not inherit resistance ($\alpha$) and the mosquito population (N) translated into variations in ${R}_{0}$, confirming their importance in insecticide resistance transmission dynamics.

The result of a sensitivity analysis clearly illustrates how changes in the parameters translate into variations in ${R}_{0}$ and therefore confirm their importance (Fig. 1).

Upon incrementing each parameter by 10%, we observed distinct variations in the basic reproduction number (${R}_{0}$). The natural birth rate ($r$) exhibited a direct relationship with ${R}_{0}$, where a 10% increase in $r$ led to a 2.47% rise in ${R}_{0}$, affirming the parameter’s positive correlation with the potential for IR transmission. Similarly, an escalation in the natural rate of resistance ($\gamma$), quantified by a similar 10% upsurge, resulted in a 2.47% increase in ${R}_{0}$, underscoring the influence of resistance inheritance on the spread of resistance. Conversely, an enlarged mosquito population (${\text{N}}$), expected to dilute the transmission rate per individual, inversely affected ${R}_{0}$. A 10% augmentation in N translated to a 4.65% reduction in ${R}_{0}$, aligning with the theory that larger populations may reduce the individual's contribution to resistance transmission. The influence of the natural death rate ($\mu$) on ${R}_{0}$ was markedly evident; an enhanced $\mu$ by 10% diminished ${R}_{0}$ by approximately 6.90%, confirming the parameter's negative correlation with ${R}_{0}$. The insecticide-induced mortality rate ($\mu i$) similarly impacted ${R}_{0}$, with a 10% increase causing a 1.64% decrement, echoing the importance of insecticide effectiveness in curbing the number of infectious vectors. Moreover, the rate of resistance acquisition through transmission ($\beta$) proved to be a critical factor. A 10% increment in $\beta$ incurred a 4.88% increase in ${R}_{0}$, highlighting its substantial role in the propagation of resistance within the mosquito population. These sensitivities are numerically supported by the Partial Rank Correlation Coefficient (PRCC) analysis (Fig. 1), yielding a PRCC of 0.85958 for $\mu$ with a significant *p*-value < 0.001, (Table S1) suggesting a strong positive association with ${R}_{0}$. On the other hand, the mosquito population size (N) had a PRCC of − 0.73351, indicating a strong negative association with ${R}_{0}$ (*p*-value < 0.001(Table S1)).

### Spatial model and validation

**Figure 2:**
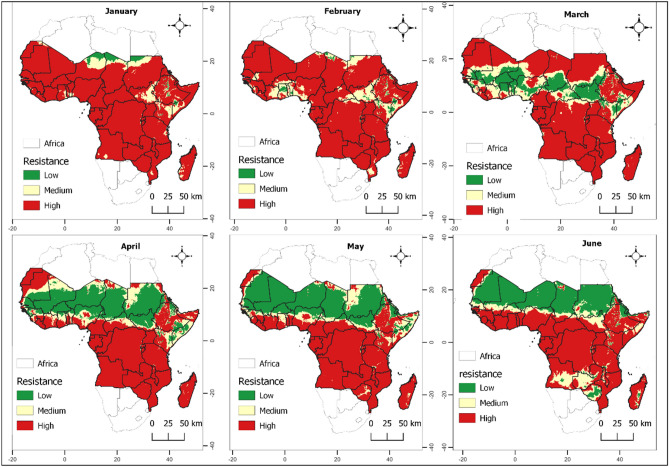
Monthly spatial mapping of the variation in ${R}_{0}$ as a function of temperature and rainfall using real data reflecting gradual insecticide resistance risk in mosquito population represented from green to red depending on the severity. Specifically low (< 1), moderate (1–1.1) and high development probability (> 1.1). The maps spanned from January 2022 to June 2022.

**Figure 3:**
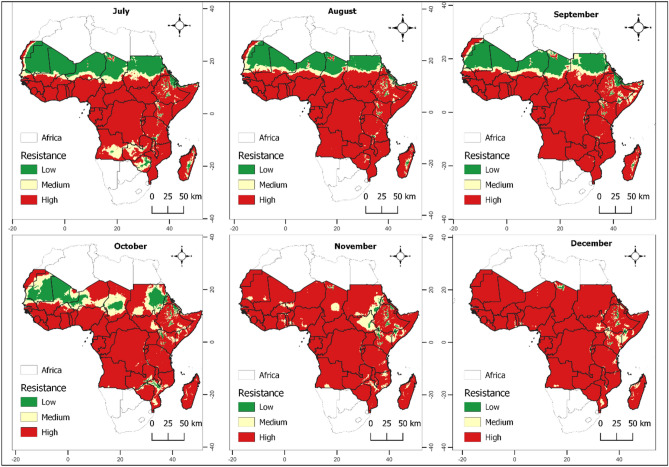
Monthly spatial mapping of the variation in ${R}_{0}$ as a function of temperature and rainfall using real data reflecting gradual insecticide resistance risk in mosquito population represented from green to red depending on the severity. Specifically, low (< 1), moderate (1–1.1) and high probability (> 1.1). The maps spanned from July 2022 to December 2022.

The data presented in Figs. 2 and 3 provide a comprehensive visualization of the monthly fluctuations of insecticide-resistant individuals among the population of mosquitoes from generation to generation in malaria-prone regions of Africa throughout 2022, drawn from actual climatic data. To ease the visualization, we categorize the outputs into low, medium, and high risk areas. These figures specifically offer a monthly geospatial delineation of ${R}_{0}$ variance, signifying the mean number of resistant mosquitoes originating from a single resistant specimen during its lifespan, subject to temperature and rainfall variations. Areas marked in red each month correspond to zones where the ${R}_{0}$ is high (over 1.1), indicating a higher likelihood of persistent number of individuals with insecticide resistance in the mosquito population. On the contrary, green zones exhibit a lower probability (under 1) of ongoing insecticide resistance within the mosquito population. Zones demarcated in yellow depict a moderate probability (between 1 and 1.1) of continued insecticide resistance propagation among individuals in the mosquitoes population.

Our investigation found significant spatial heterogeneity in the population of insecticide-resistant individuals, varying considerably across countries, regions, and even seasons. An uptick in the likelihood of insecticide resistance within mosquito populations is more likely in the lower regions of sub-Saharan Africa during the initial quarter of the year when temperature and precipitation are high. A parallel pattern was noticed in the northern sub-Saharan African nations during October to December. The spatial mapping of the population of insecticide-resistant mosquitoes closely correlates with regions known for high malaria incidence areas (as displayed in Figs. 2 and 3). These findings contribute a reliable approach for evidence-based resistance management strategies through incorporating of climate data that mosquito populations are exposed to in real ecosystem settings.

**Figure 4:**
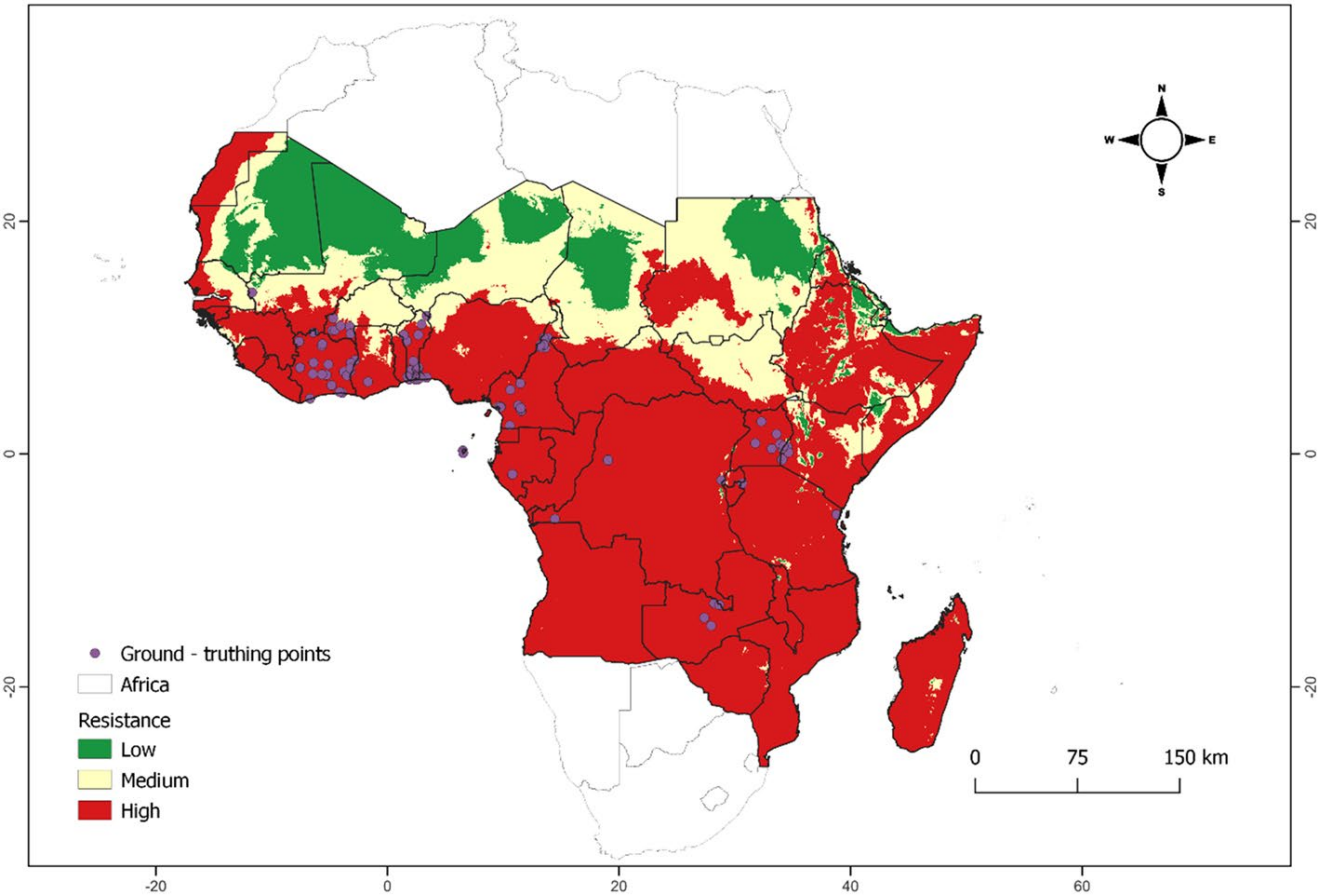
Validation of the basic reproduction number (${R}_{0}$) using spatial overlay by comparing the predicted annual model outputs with empirical ground truthing data of insecticide resistance individuals in the mosquitoes population.

Figure 4 illustrates the validation approach using spatial overlay. Practically ${R}_{0}$ indicates the likelihood that a single resistant mosquito can rise in the population during its lifetime. ${R}_{0}$ < 1 denotes the likelihood of the propagation of insecticide resistance in the population is low or improbable. Moderate i.e. ${R}_{0}$ slightly greater or equal to 1 (${R}_{0}$ > 1) denotes a moderate likelihood of the persistence of insecticide resistance in the mosquito population over time whereas high ${R}_{0}$, signify high probability.

Validating the ${R}_{0}$ can be a complex task due to its interpretability. Comparing it with real-world data, serve as a useful tool for interpreting ${R}_{0}$ in real-world scenarios. Our model exhibits a high degree of realism (with over 92. 14% points falling in the high classes) with ground-truthing data, which encourages the use of the tool in the context of decision-making (Fig. 4). This implies that this model can be used under weather variability to determine insecticide resistance trends in the number of individuals within mosquito population at scale and that the underlying assumptions made based on the bioecology of the mosquito are somehow realistic.

### Projection of the basic reproduction number (${{\varvec{R}}}_{0}$) at scale

**Figure 5:**
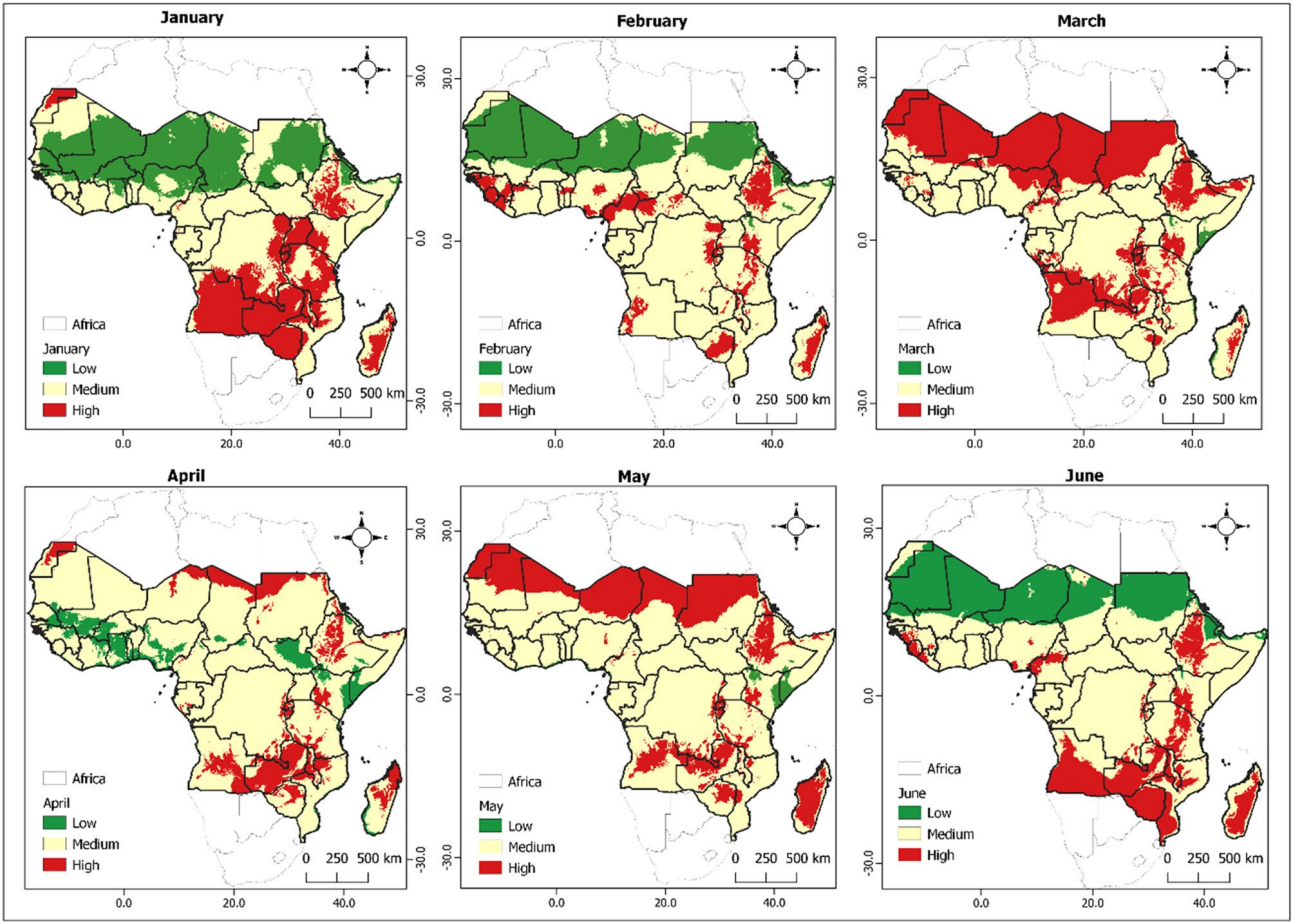
Monthly spatial projection of the variation in ${R}_{0}$ as a function of temperature and rainfall reflecting gradual insecticide resistance risk in mosquito population represented from green to red depending on the severity. Specifically low (< 1), moderate (1–1.1) and high development probability (> 1.1). The maps spanned from January 2023 to June 2023.

**Figure 6:**
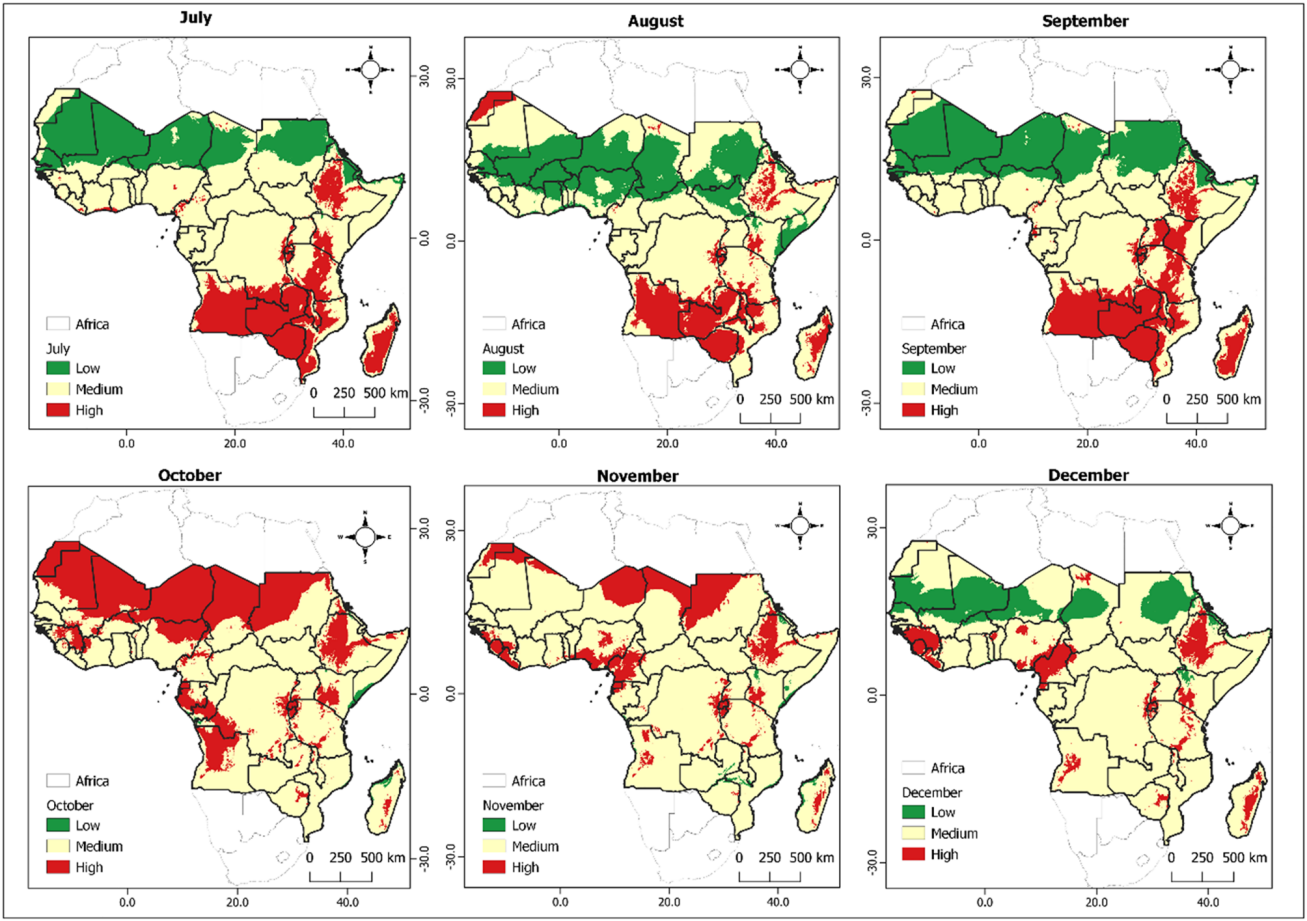
Monthly spatial projection of the variation in ${R}_{0}$ as a function of temperature and rainfall reflecting gradual insecticide resistance risk in mosquito population represented from green to red depending on the severity. Specifically, low (< 1), moderate (1–1.1) and high probability (> 1.1). The maps spanned from July 2023 to December 2023.

Figures 5 and 6 display the monthly variation of the number of individuals within the mosquito population with insecticide resistance in malaria-risk areas in the African landscape using projected data for the year 2023. This projections aligned well with the real data showing the study areas at a high risk of insecticide resistance persistence in the mosquito population and therefore validating the robustness of the model. We also tested the accuracy procedure on the projected data and observed. The projected model exhibits a reasonable degree of realism (with over 63. 50% points falling in the high classes considering no drastic changes when transiting from 2022 to 2023).

## Discussion

In this study, we developed a mathematical model to investigate the spatial trend of insecticide resistance in *An. gambiae* populations under the influence of climate variability at a fine geographical scale. The model specifically demonstrates that when insecticide resistance emerges within a mosquito population, it either propagates across generations or eventually disappears. Our model adapted a SIR framework with environmental factors with very good accuracy (> 92% and 63% respectively for real and projected data), such as temperature and rainfall, to capture the intricate relationships between mosquito population dynamics, individuals with insecticide resistance, and climate conditions. The model focuses on the interactions between susceptible mosquitoes (S), resistant mosquitoes (R), and non-resistant offspring (M). It accounts for various factors such as birth rate, natural death rate, and insecticide-induced mortality rate, as well as the transmission and recovery rates from insecticide resistance.

Our model diverges from the traditional frameworks by focusing not just on immediate vector control outcomes but on the long-term persistence and spread of resistance across generations. This broader perspective complements existing compartmental models, such as those discussed by Wairimu et al.^39^, which emphasize the critical role of the control reproduction number and the efficacy of control strategies in mitigating the effects of insecticide resistance. Similarly, the work by Ratti et al.^40^, which employs a detailed model to evaluate the impact of indoor wall treatments, informs our understanding of the temporal suppression of vector populations. More importantly, the contributions of Mohammed-Awel et al.^41^, present a novel deterministic model that enriches the analytical landscape by incorporating a stratification of mosquito populations based on resistance type and feeding preferences. This stratification allows for a nuanced exploration of the population-level impacts of mosquito insecticide resistance, extending beyond the scope of singular intervention strategies like IRS or ITNs alone. Our model extends these discussions by offering a generic form that assesses the overarching trends of insecticide resistance spread in response to climatic influences. Importantly, our approach underscores the necessity of considering the evolutionary dynamics of resistance, highlighting that the mere eradication of resistant mosquitoes may not suffice. Instead, our model illustrates that understanding the conditions under which resistance might either persist or diminish is crucial for devising effective and sustainable malaria control strategies. By doing so, it fills a critical gap in the current modeling literature, which often focuses on specific interventions without fully accounting for the broader evolutionary and ecological dynamics of resistance.

Our findings highlight the advantages of the SIR model over the data-driven model especially when the target is a general framework of the transmission mechanism. Specifically, the SIR model offers a mechanistic comprehension of the fundamental processes involved, including transmission, recovery, and resistance dynamics^38^. Moreover, we identified key parameters, like transmission and recovery rates, that drive the proportion of individuals with insecticide resistance^37^. For example, the results of sensitivity analysis of the model revealed that perturbation of model parameters (the natural birth rate of mosquitoes ($r$), the natural rate of resistance ($\gamma$), the transmission rate ($\beta$), the natural death rate of mosquitoes ($\mu$), insecticide-induced mortality rate ($\mu i$), and the mortality rate due to offspring of resistant mosquitoes that do not inherit resistance ($\alpha$)) mirrored variations in ${R}_{0}$, confirming their importance in proportion of the number of individuals within the mosquito population with insecticide resistance and the transmission dynamics^42^.

The current study, focusing on the intricate relationship between climate variables, specifically temperature and rainfall, and the development and spread of insecticide resistance, aligns with and builds upon the conclusions drawn by Worrall et al.^43^. They highlighted how climatic conditions significantly influence malaria transmission rates, which by extension, affect the emergence and proliferation of insecticide resistance. Worrall et al.^43^’s work shed light on the critical role of environmental factors in modulating vector biology and malaria epidemiology, suggesting that shifts in rainfall and temperature patterns could directly impact the effectiveness of vector control strategies and the dynamics of resistance evolution. Echoing Worrall et al.^43^, our findings reveal that regions experiencing pronounced fluctuations in temperature and rainfall are particularly susceptible to the accelerated development and spread of insecticide resistance. This correlation underscores the necessity of integrating climate data into the planning and execution of vector control interventions. By doing so, we can better anticipate areas at risk of heightened IR potential and tailor our strategies accordingly, ensuring they remain effective under changing climatic conditions. Our application of 95% confidence interval (CI) maps further emphasizes the spatial correlation between climatic variability and IR hotspots, providing a visual tool for identifying areas where focused intervention may be most needed.

An intriguing pattern our study has revealed is the surge in insecticide resistance within mosquito populations in the lower regions of sub-Saharan Africa during the first quarter of the year. This temporal pattern has important implications for the planning of insecticide interventions. The simultaneous observation of a similar pattern in the southern African countries during June and July further underscores the necessity of tailoring interventions to the unique temporal and spatial patterns observed. The strong correlation between the spatial mapping of insecticide-resistant mosquitoes and areas with a high incidence of malaria (illustrated in Figs. 2 and 3) validates the relevance of our research. This relationship suggests that an increase in insecticide resistance may directly contribute to the persistence of malaria in these regions.

Moving forward, understanding these resistance patterns could enable more effective targeted control of insecticide-resistant mosquitoes, potentially improving malaria prevention efforts. As the spatial and temporal trends of insecticide resistance continue to evolve, it will be essential to maintain ongoing surveillance and research to inform adaptive and effective strategies for malaria control. This mirrors the rainfall and temperature regimes across Africa where malaria incidence is already known to be high^44^. For example, the peak month of rainfall in many West African countries ie., August or September, during the middle to late part of the rainy season^45^. In Hancock et al.^2,3^ climate variability was considered to play an important role in insecticide resistance transmission dynamics, and consequently, in insecticide resistance management. In particular, temperature and rainfall significantly influenced mosquito population growth rates^46,47^ affecting the propagation of insecticide resistance in mosquito populations. In addition, the study demonstrated that areas with high climate variability should experience a more rapid and widespread dissemination of insecticide resistance, which has important implications for vector control strategies in these regions^44^. This reinforces the fact that climate condition in Africa favors the reproduction and proliferation of mosquito vectors^48^, therefore the intensive utilization of insecticide leads to the rapid development of the number of individuals within the mosquito population with insecticide resistance. Hence the rapid development of resistant mosquitoes within the population compromises the effectiveness of current vector control measures, such as insecticide-treated nets and indoor residual spraying^49^. This highlights the need for adaptive and integrated vector control strategies that take into account the spatial patterns of insecticide resistance and climate variability.

Although our study provides valuable insights into the interactions between mosquito population dynamics, insecticide resistance, and climate conditions, and was validated using empirical data, the study has several limitations that warrant further research. First, the model is very simple and could be refined by incorporating additional environmental factors, such as humidity and land use, which can also influence mosquito population dynamics hence the propagation of insecticide resistance^50^. However, incorporating such parameters would necessitate sourcing empirical data on how the insecticide resistance vary under changing humidity and land use, especially in the contexts of agricultural practices that may influence development of resistance. Finally, future research could explore the potential impact of novel vector control tools, such as gene drive technology^51–53^ and new insecticides^54^, as well as *Microsporidia MB*^22,55^ on the impact on the number of individuals with resistance features within the mosquito population. Additional research can enhance the current findings by incorporating the birth rate of non-resistant mosquito offspring (M) within the resistant class, which can determine the transmission rate (α) for susceptible offspring of resistant mosquitoes. This extension could bring new insights and significant benefits in understanding the mechanism underpinning insecticide resistance propagation in mosquito populations globally. Also, it is crucial to emphasize that the mathematical model developed in this study is founded on logical reasoning and basic knowledge of the biomechanisms involved in the propagation of insecticide resistance in the mosquito population.

The study does not inherently fall under any specific class of insecticides. Instead, it provides a general framework for studying the dynamics of insecticide resistance transmission in mosquito populations, which can be adjusted and refined to study specific classes of insecticides. Ultimately, the model presented here serves as a generic and valuable tool for use by decision-makers to design and implement effective, context-specific vector control measures that can adapt to the changing dynamics of the number of resistant mosquitoes in a population to climate variability. The study underscores the importance of considering climate variability in modeling the propagation of insecticide resistance in *An. gambiae* populations. The mathematical model presented here provides a valuable tool for understanding the complex interactions between mosquito population dynamics, insecticide resistance, and climate conditions, and can inform the design and implementation of more effective, context-specific vector control measures. By addressing the challenges posed by insecticide resistance under climate variability, this research contributes to the global effort to combat malaria and safeguard the well-being of millions of people at risk of this life-threatening disease.

In conclusion, our study presented a mathematical model to examine the trend of insecticide resistance in *An. gambiae* populations under the influence of climate variability at a regional scale. By integrating a SIR framework with environmental factors such as temperature and rainfall, we better understood the intricate relationships between mosquito population dynamics, insecticide resistance, and climate conditions. Our findings underscored the importance of considering climate variability when modeling the propagation of insecticide resistance among mosquito populations. The results demonstrated the significant impact of temperature and rainfall on mosquito population growth rates and the dynamic of the number of individuals within the mosquito population with insecticide resistance. These findings can help national malaria control programmes to focus the surveillance of insecticide resistance in targeted areas thereby reducing the area of surveys. Overall, this highlights the need for adaptive and integrated vector control strategies that account for the patterns of the number of individuals within the mosquito population with insecticide resistance and climate variability.

## Methods

### Study area and datasets used

#### Study area

The study area covers high malaria risk area within the Africa continent (https://www.wanda.be/en/a-z-index/malaria-kaart-afrika-/) where ideal climate favors the growth, reproduction and proliferation of mosquito vectors^48^. Rainfall and temperature are critical environmental factors that influence mosquito population growth rates. They can affect various aspects of mosquito life history, including development, survival, reproduction, and overall population dynamics^56,57^. The model assumes that temperature and rainfall are the key factors affecting the increase in the number of individuals within the mosquito population with insecticide resistance. Therefore, other environmental variables that can influence the number of individuals within the mosquito population with insecticide resistance are assumed to be set optimal. To understand the relationship between the number of individuals within the mosquito population with insecticide resistance and climatic conditions, we used freely available high-resolution climatic data.

#### Resistance dataset

Our analysis incorporates data on insecticide resistance status among mosquito populations, an essential factor in understanding and validating resistance spread in the population. The “resistance status” refers to the genetic propensity of mosquito populations to withstand insecticidal control measures, categorized into “resistant” and "susceptible" groups. This categorization is based on bioassay test results, which assess the mortality rates of mosquito samples exposed to standard doses of insecticides. Our validation dataset predominantly focuses on *An. gambiae*, known vectors of malaria, with resistance status aggregated from multiple sources, including peer-reviewed studies and reports from national malaria control programs sourced from the World Health Organization (WHO) Threat website (Malaria Threat Map (who.int)). For simplicity, we assume that only pyrethroid-related interventions, e.g., ITN, IRS, and mosquito-repellents are used in the study area. The data comprise a total of 394 observations spanning from 2000 to 2019. The data on resistance status primarily pertains to female *An. gambiae* mosquitoes, as females, are the vectors for malaria transmission. They are the ones that feed on human blood and, consequently, are targeted in vector control strategies, including the use of insecticides. This focus aligns with the operational needs of malaria control and elimination strategies, where understanding and mitigating resistance in female mosquitoes is crucial for reducing malaria transmission. Out of 394 records spanning 2000 to 2019, we selectively employed 295 confirmed cases of resistance (Instances of insecticide resistance in Anopheles mosquitoes that have been rigorously verified through standardized bioassay tests as per World Health Organization (WHO) guidelines), ensuring robust model validation. Given the uneven spatial distribution of these data points, we treated the dataset holistically to validate our model across diverse geographic settings.

#### Climatic data sources

For our analysis, we employed high-resolution annual temperature and rainfall raster data obtained from TerraClimate, accessed via Google Earth Engine^58^. TerraClimate provides a comprehensive dataset of monthly climate and climatic water balance for global terrestrial surfaces, serving as a reliable source for our model inputs^59^. To project insecticide resistance trends for 2023, we utilized downscaled global circulation models from Envidat, offering monthly precipitation sums and average temperatures. This data, with approximately 5km spatial resolution covering the period 1850–2100^60^, enables us to simulate future resistance scenarios under varying climatic conditions. The rationale behind utilizing the mean temperature in our analysis is twofold. First, it provides a simplified yet effective measure of the thermal environment, reflecting average conditions under which mosquito populations thrive or decline. Second, mean temperature data is widely used in vector-borne disease modeling to approximate the thermal suitability for vector population dynamics over time. This approach allows us to assess the potential impact of climatic variables on the geographical distribution and seasonal patterns of mosquito populations and associated resistance trends.

### Mathematical model

#### Model formulation

In the formulation of our mathematical model, while we do not explicitly disaggregate the mosquito population by gender, we implicitly focused on female *An. gambiae* mosquitoes. This stems from their pivotal role in malaria transmission, as female mosquitoes are the primary vectors through their blood-feeding behavior necessary for the malaria parasite's life cycle. Specifically, we targeted a model that demonstrates when insecticide resistance emerges in a number of individuals within the *An. gambiae* African mosquito population, and how it either propagates across generations or eventually dies out. The mathematical model to study the number of individuals within the mosquito population with insecticide resistance was formulated using a system of non-linear ordinary differential equations to be as simple and reproducible as possible. We defined three compartments to represent the different states of resistance within the population:

1. Susceptible (S): Mosquitoes that are not resistant to insecticides.
2. Resistant (R): Mosquitoes that are resistant to insecticides.
3. Non-resistant offspring (M): Offspring of resistant mosquitoes that do not inherit resistance.

**Figure 7:**
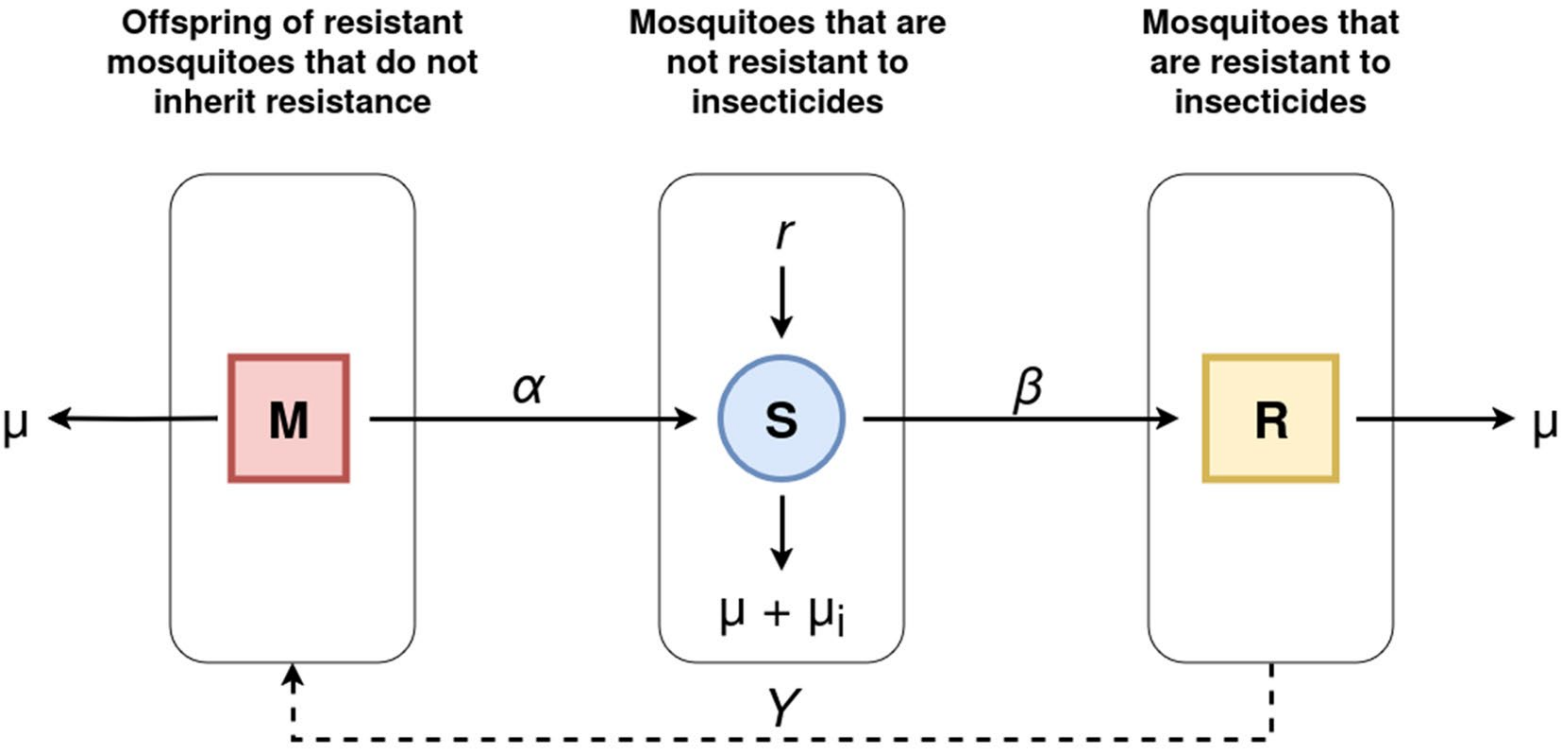
Diagrammatic of the proposed model showing the three compartments that represent the different states of resistance within the population of mosquitoes.

Figure 7 shows the diagram of the proposed model showing the three compartments that represent the different states of resistance within the population with Susceptible (S): Mosquitoes that are not resistant to insecticides; Resistant (R): Mosquitoes that are resistant to insecticides and Non-resistant offspring (M): Offspring of resistant mosquitoes that do not inherit resistance.

The following model parameters were considered when defining and solving the mathematical problem:

1. $\beta$: The transmission rate is defined as the probability that a mosquito becomes resistant due to contact with an insecticide.
2. $\gamma$: The recovery rate is defined as the probability that a resistant mosquito produces non-resistant offspring.
3. $\alpha$: is defined as the rate at which offspring of resistant mosquitoes that do not inherit resistance become susceptible again.
4. $\mu$: represent the natural death rate of mosquitoes.
5. $\mu i$: represent the insecticide-induced mortality rate
6. $r$: represent the birth rate of mosquitoes.

For spatial simulation, we assumed that only pyrethroid-related interventions (e.g. ITN, IRS, and mosquito-repellents) were used, and that about 3–5% of the infectious mosquitoes develop resistance as reported in Bøgh et al.^61^. Therefore, $\beta$ is at least 50%. Moreover, Torres et al.^62^ demonstrated that the probability of an *An. gambiae* resistant mosquito to produce a non-resistant offspring depends on the specific resistance gene and the mode of inheritance. For example, two resistant mosquitoes with homozygous knockdown resistance (*kdr*) mutation mating, will result in all their offspring inheriting the resistance allele and therefore be resistant (vertical transmission is perfect). In other conditions, only 25% are expected to be homozygous resistant. Hence there is at least a probability that a resistant mosquito produces non-resistant offspring.

Our approach provides two modelling scenarios (model 1 and model 2) whereby model 1, represents the case where the transmission is considered imperfect as a percentage of non-resistant offspring (M) transition to Susceptible (S) mosquitoes at the defined rate $\alpha$. For model 2, transmission is considered perfect (100%) hence there is no transition of non-resistant offspring (M) to Susceptible (S) mosquitoes and $\alpha$ will be equal to zero. The following assumptions were considered to model the transition of non-resistant offspring (M) to Susceptible (S) mosquitoes:

##### Assumptions made for model 1

- N is the initial mosquito population (Eqs. 1 and 2).
- The transmission rate $\beta$ depends on the contact rate between susceptible mosquitoes’ and insecticide, and the probability of transmission per contact.
- The mortality rate due to insecticide application ${\mu }_{i}$ depends on the frequency of insecticide application, the specific insecticide used, and the concentration applied and is assumed to be constant and proportional to the number of resistant mosquitoes.
- The natural mortality rate $\mu$ vary according to temperature as proposed by Parham & Michael^57^ (Eq. 13)
- The rate at which offspring of resistant mosquitoes that do not inherit resistance become susceptible at the rate of α is assumed to be constant.
- α exist for the imperfect transmission vertical in the *An. gambiae* mosquitoes population.

The formulation of model 1 is as follows:

1 $$\left\{\begin{array}{c}\frac{dS}{dt}=r-\left(\mu +{\mu }_{i}\right)S-\frac{\beta }{N}SR-\gamma SM+\alpha MS\\ \frac{dR}{dt}=\frac{\beta }{N}SR-\mu R\\ \frac{dM}{dt}=\gamma SM-\alpha MS-\mu M\end{array}\right..$$

##### Assumptions made for model 2

The assumptions of the mosquito populations are the same as in model 1. Model 2 considers the interaction between Susceptible (S) mosquitoes that are not resistant to insecticides and non-resistant offsprings (M) of resistant mosquitoes that do not inherit resistance. The underlying assumption of model 2 is that α does not exist for the vertical transmission in the mosquito population at 100%. Herein, when α = 0, model 1 translates to an extension of model 2. The equation of model 2 is as follows:

2 $$\left\{\begin{array}{c}\frac{dS}{dt}=r-\left(\mu +{\mu }_{i}\right)S-\frac{\beta }{N}SR-\gamma SM\\ \frac{dR}{dt}=\frac{\beta }{N}SR-\mu R\\ \frac{dM}{dt}=\gamma SM-\mu M\end{array}.\right.$$

### Resistance-free equilibrium point (RFE)

In this study, the disease-free equilibrium, denoted the resistance-free equilibrium (RFE) in the context of model (1), which represents a state where the population does not contain any individuals with insecticide resistance. It is an equilibrium point where the population variables reach a stable state. Specifically, the RFE corresponds to a set of values for the population variables that result in zero (0) prevalence of insecticide resistance. To determine this equilibrium point, we calculate the derivatives of the variables and them equal to zero:

3 $$\left\{\begin{array}{c}\frac{dS}{dt}=0\\ \frac{dR}{dt}=0\\ \frac{dM}{dt}=0\end{array}\right..$$

4 $$\mathrm{This\,\, gives\,\, us \,\,the \,\,following\,\, system \,\,of \,\,equations}: \left\{\begin{array}{c}r-\left(\mu +{\mu }_{i}\right){S}_{0}-\frac{\beta }{N}{S}_{0}{R}_{0}-\gamma {S}_{0}{M}_{0}+\alpha {M}_{0}{S}_{0}=0\\ \frac{\beta }{N}{S}_{0}{R}_{0}-\mu {R}_{0}=0\\ \gamma {S}_{0}{M}_{0}-\alpha {M}_{0}{S}_{0}-\mu {M}_{0}=0\end{array}.\right.$$

From the first equation, we have,

5 $$r-\left(\mu +{\mu }_{i}\right){S}_{0}-\frac{\beta }{N}{S}_{0}{R}_{0}-\gamma {S}_{0}{M}_{0}+\alpha {M}_{0}{S}_{0}=0.$$

At the resistance-free equilibrium, there are no resistant mosquitoes due to the insecticide (R = 0), there are no Offspring of resistant mosquitoes (M = 0) and the number of susceptible mosquitoes S is equal to the birth rate *r* divided by the total mortality rate of susceptible mosquitoes (natural mortality rate + mortality rate due to insecticide application and resistance). Moreover, the number of susceptible and resistant mosquitoes is not related to transmission, as there is no transmission of insecticide resistance. Consequently, if ${R}_{0}={M}_{0}=0$ then,

6 $$r-\left(\mu +{\mu }_{i}\right){S}_{0}-\frac{\beta }{N}{S}_{0}(0)-\gamma {S}_{0}(0)+\alpha (0){S}_{0}=0,$$

7 $$r-\left(\mu +{\mu }_{i}\right){S}_{0}=0, {S}_{0}=\frac{r}{\left(\mu +{\mu }_{i}\right)}.$$

Therefore, the resistance-free equilibrium is given by the following equation:

8 $${E}^{0}\left({S}_{0}, {R}_{0}, {M}_{0}\right)={E}^{0}\left({S}_{0}, 0, 0\right)={E}^{0}\left(\frac{r}{\left(\mu +{\mu }_{i}\right)},0, 0\right).$$

### Basic reproduction number ${{\varvec{R}}}_{0}$

The basic reproduction number (${R}_{0}$) represents the average number of resistant mosquitoes that a single resistant mosquito can give rise to in its lifetime. This number describes the propagation of insecticide resistance within the mosquito population from one generation to another. In model 1 (Eq. 1), we calculated the basic reproduction number as the spectral radius of the next generation matrix. In this case, the next generation matrix is a square matrix that describes the expected number of new resistant cases in each population for every resistant individual.

To calculate the basic reproduction number, ${R}_{0}$, we estimated the spectral radius of the next-generation matrix.

The next-generation matrix for the model 1 is:

9 $$\genfrac{}{}{0pt}{}{\begin{array}{c}\frac{r}{\left(\mu +{\mu }_{i}\right)}\\ 0\end{array}}{0} \genfrac{}{}{0pt}{}{\begin{array}{c}\frac{\beta }{N}{S}_{0}\\ \frac{\beta }{N}{S}_{0}+\mu +\gamma \end{array}}{\gamma {S}_{0}} \genfrac{}{}{0pt}{}{\begin{array}{c}\alpha {M}_{0}\\ 0\end{array}}{\alpha {S}_{0}+\mu }.$$

The spectral radius is the maximum eigenvalue of this matrix. The characteristic equation of the matrix is given by:

10 $$G=\genfrac{}{}{0pt}{}{\begin{array}{c}\frac{r}{\left(\mu +{\mu }_{i}\right)}-\lambda \\ 0\end{array}}{0} \genfrac{}{}{0pt}{}{\begin{array}{c}\frac{\beta }{N}{S}_{0}\\ \frac{\beta }{N}{S}_{0}+\mu +\gamma -\lambda \end{array}}{\gamma {S}_{0}} \genfrac{}{}{0pt}{}{\begin{array}{c}\alpha {M}_{0}\\ 0\end{array}}{\alpha {S}_{0}+\mu -\lambda }.$$

The ${R}_{0}$ is the spectral radius (maximum eigenvalue) of matrix G:

11 $${R}_{0}=\frac{1}{\mu +{\mu }_{i}+\alpha }\sqrt{\frac{\beta (r+\gamma )}{N}.}$$

If ${R}_{0}$ > 1, insecticide resistance will propagate in the mosquito population. If ${R}_{0}$ < 1, the insecticide resistance will die out over time.

Furthermore, the main assumption of this study is that mosquito birth rate $r$ depends on rainfall, a parameter that influences breeding site availability, and also temperature for survival^57^. Temperature and rainfall dependence aspect of ${R}_{0}$ was formulated in Eq. (11). Specifically, the mosquito birth rate and death rate formulated by Parham & Michael^57^ as a function of temperature and rainfall, were incorporated into Eq. (11) as the natural birth rate of the mosquitoes. The obtained ${R}_{0}$ is as follows:

12 $${R}_{0 }= \frac{1}{\mu \left(T\right)+{\mu }_{i}+\alpha }\sqrt{\frac{\beta \left(r\left(R,T\right)+\gamma \right) }{N}},$$

13 $$\mu \left(T\right) = \frac{1}{a{T}^{2}+bT+c},$$

14 $$r\left(R,T \right) = {B}_{E}{\theta }_{E}{\theta }_{L}{\theta }_{p}{\left(\frac{4R\left({R}_{L}-R\right)}{{R}_{L}^{2}}\right)}^{3} \frac{\left({k}_{1}T+{k}_{2}\right){e}^{-\left({k}_{1}T+{k}_{2}\right)}}{2\left({c}_{1}T+{k}_{2}\right)+1},$$

15 $${R}_{0}=\sqrt{\frac{\beta \left({B}_{E}{\theta }_{E}{\theta }_{L}{\theta }_{p}{\left(\frac{4R\left({R}_{L}-R\right)}{{R}_{L}^{2}}\right)}^{3} \frac{\left({k}_{1}T+{k}_{2}\right){e}^{-\left({k}_{1}T+{k}_{2}\right)}}{2\left({c}_{1}T + {k}_{2}\right)+1} +\gamma \right){\left(a{T}^{2}+bT+c\right)}^{2}}{N{\left(\left(a{T}^{2} +bT + c\right) \left({\mu }_{i}+\alpha \right) + 1\right)}^{2}}},$$

### Sensitivity analysis of the model parameters on ${{\varvec{R}}}_{0}$

A sensitivity analysis was conducted to evaluate the extent to which the model output ${R}_{0}$ (Eq. 10) is affected by variations in the model parameters. This analysis measures the degree to which changes in the parameters result in fluctuations in ${R}_{0}$ and provides an assessment of the relative significance of various factors contributing to the transmission potential of insecticide resistance within a number of individuals in the mosquito population. We performed the sensitivity analysis using the Partial Rank Correlation Coefficient (PRCC) algorithm^63^, where the greater or smaller PRCC values signify that the parameter has a strong or fair impact on the model outcome whether positive or negative (Table S1 Supplementary). A positive PRCC value indicates that an increase in the parameter leads to insecticide resistance propagation increase, while a negative value suggests that an increase in the parameter results in a decrease in insecticide resistance propagation. The PRCC was implemented in Matlab software^64^. In addition, we incremented and decremented each parameter by 10% from their baseline values and observed the resulting variation in ${R}_{0}$. The baseline value of ${R}_{0}$ was computed to be 0.02357(Table S2 Supplementary).

### Model and performance assessment (validation)

To assess the model performance in predicting the transmission potential of insecticide resistance to a number of individuals within a mosquito population, we divided the area of study (Africa) into matrices or grids. We determined the coordinates of the centroid of each grid and extract the monthly average value of the temperatures and rainfall of the year 2022 from the available datasets using Google Earth engine^58^ and then, estimated the value of ${R}_{0}$ with Eq. (15) in each grid. The resulting value of ${R}_{0}$ obtained in each grid is reconverted into American Standard Code for Information Interchange (ASCII) files through spatial interpolation performed with Quantum-Geographical Information System (Q-GIS version 3.10)^65^, which further served for visualization and mapping.

In addition to generating 12 monthly ${R}_{0}$ raster maps, our methodology included calculating the 95% confidence interval (CI) for ${R}_{0}$ to assess spatial variability and uncertainty. Using R's “raster**”** package^66^, we computed the mean and standard deviation across the monthly maps and then derived the 95% CI, producing a comprehensive spatial representation (Fig. S Supplementary). This process generated two additional rasters representing the lower and upper bounds of the 95% CI for ${R}_{0}$, thereby offering a visual depiction of uncertainty and variability in mosquito population dynamics influenced by environmental factors. To calculate the 95% CI, we applied the formula:

16 $$CI=Mean\pm \left(1.96\times SD\right),$$

where "Mean" represents the average ${R}_{0}$ value for each pixel across the 12 monthly rasters, and "SD" is the standard deviation at each pixel, indicating the variability of ${R}_{0}$.

Subsequently, the areas where pyrethroid resistance was observed during data collection period (2000–2019) are overlaid on top of a generated spatial projection of the basic reproduction number (${R}_{0}$) (Eq. 15) using a 50% annual contribution of the current monthly temperature and rainfall of the year 2022. The obtained model is evaluated based on its ability to accurately represent field data through visualization.

### Projection of the basic reproduction number (${{\varvec{R}}}_{0}$)

The model Eq. (15) was run monthly for the year 2023 to provide a potential state of projected situation of insecticide resistance potential trends in the malaria risk areas of Africa. In this context, we inputted the value of projected temperatures and rainfall (Envidat) corresponding to the geographical coordinates the centroid of the grids presented above. The mapping process follows the same procedure as described earlier. In the expression of (${R}_{0}$) as a function of temperature and rainfall other parameters for Eq. (15) are provided in Table 1.

### Supplementary Information

Supplementary Information.

## Data Availability

The datasets used and/or analysed during the current study available from the corresponding author on reasonable request.
