# Supplementary material for: A mathematical model for mapping the insecticide resistance trend in the Anopheles gambiae mosquito population under climate variability in Africa
Source: Sci Rep. 2024 Apr 29;14:9850. doi: 10.1038/s41598-024-60555-z (PMC11059405; doi:10.1038/s41598-024-60555-z)
Supplement: Supplementary file 1 — Supplementary Information. [file 41598_2024_60555_MOESM1_ESM.docx]

**Supplementary**

**95 % Confidence interval map**

Subtracting the upper bound CI raster from the lower bound CI raster provides a useful map of the width of the confidence interval across your study area, which reflects the uncertainty in the $R_{0}$​ estimates.

**
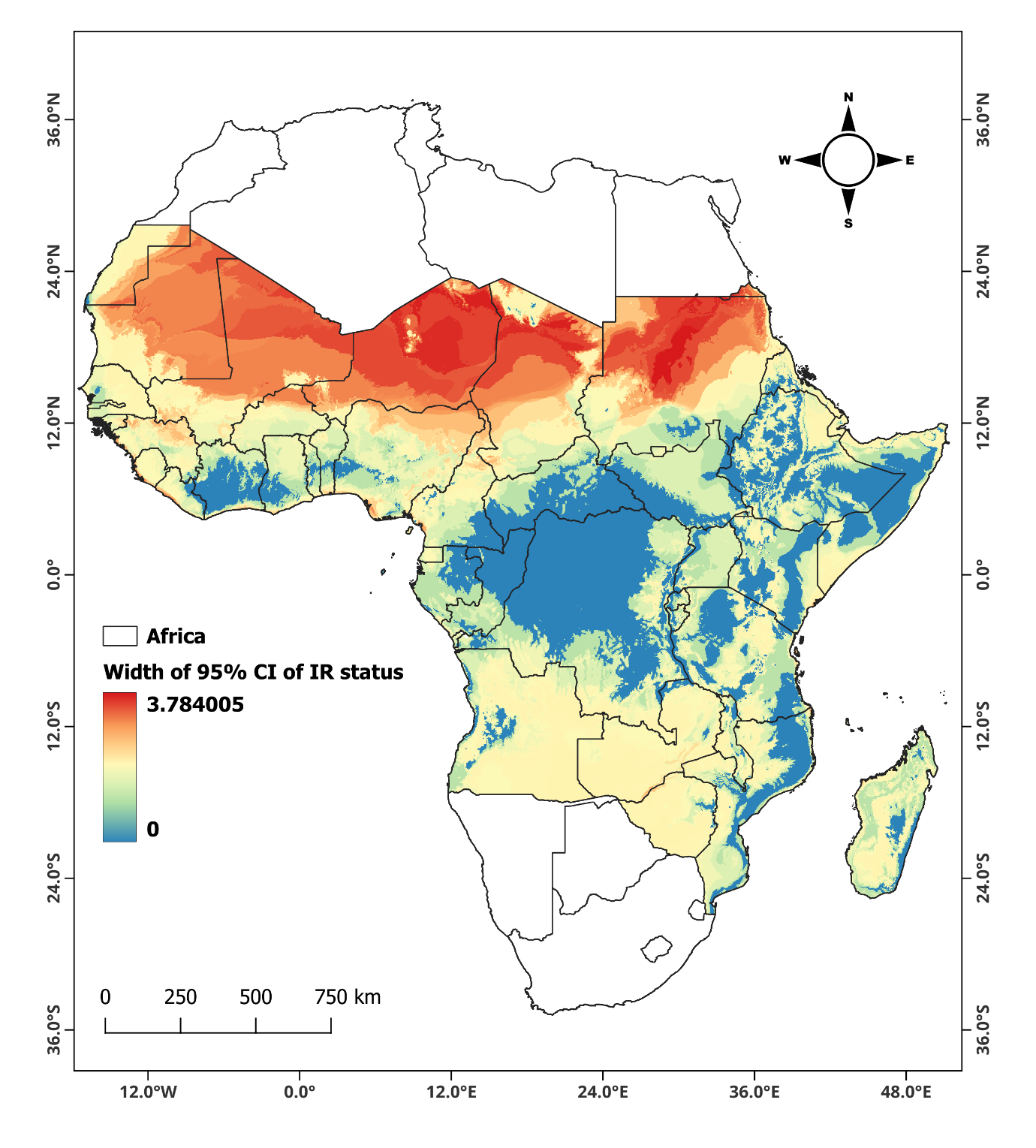
**

**Figure S.** Annual variability in insecticide resistance $R_{0}$ confidence interval width across the study area**.** Map depicting the annual variability in the width of the 95% confidence interval (CI) for insecticide resistance $R_{0}$​ estimates derived from monthly data across the study area. Higher values (represented by warmer colors) indicate greater variability in $R_{0}$​ estimates, suggesting areas or periods with significant environmental influence or data sparsity. Lower values (represented by cooler colors) signify areas or periods with more stable $R_{0}$​ estimates, potentially reflecting consistent environmental conditions or the effectiveness of vector control measures.

- **Higher values on the CI width raster**: These indicate areas with greater variability or uncertainty in insecticide resistance $R_{0}$​ estimates. Essentially, higher values mean that the difference between the upper and lower bounds of the confidence interval is larger, reflecting less certainty about the $R_{0}$​ value. This could be due to various factors such as environmental variability affecting mosquito behavior, effectiveness of insecticide interventions, or data sparsity.
- **Lower values on the CI width raster**: Conversely, lower values suggest areas where there is less variability or greater certainty in the $R_{0}$​ estimates. Smaller differences between the upper and lower confidence bounds indicate more confidence in the $R_{0}$​ estimate. This could reflect more stable environmental conditions.Top of Form

Bottom of Form

**Sensitivity analysis**

**Table S1.** PRCC values for each parameter to visualize their sensitivity influence on $R_{0}$​

| Parameter | PRCC | *p*-value |
| --- | --- | --- |
| $\boldsymbol{\mu}$ | 0.85958 | 2.3E-122*** |
| $\boldsymbol{\mu i}$ | -0.35196 | 9.37E-08*** |
| $\boldsymbol{\alpha}$ | -0.32293 | 1.76E-08*** |
| $\boldsymbol{\beta}$ | 0.70657 | 1.45E-56*** |
| $\boldsymbol{r}$ | 0.48029 | 9.32E-17*** |
| $\boldsymbol{\gamma}$ | 0.45806 | 2.14E-19*** |
| N | -0.73351 | 9.4E-22*** |

**Table S2.** Incremental of each model parameter by 10%, with observed nuanced effects on the basic reproduction number **(**$R_{0}$**​)**

| Parameter | Baseline Value | Increased Value | Decreased Value | R0 Baseline | R0 Increase | R0 Decrease | Change Increase (%) | Change Decrease (%) |
| --- | --- | --- | --- | --- | --- | --- | --- | --- |
| $\boldsymbol{\mu}$ | 0.5 | 0.55 | 0.45 | 0.02357 | 0.025090886 | 0.021944693 | 6.451612903 | -6.89655 |
| $\boldsymbol{\mu i}$ | 0.5 | 0.55 | 0.45 | 0.02357 | 0.023183829 | 0.023969721 | -1.639344262 | 1.694915 |
| $\boldsymbol{\alpha}$ | 0.5 | 0.55 | 0.45 | 0.02357 | 0.023183829 | 0.023969721 | -1.639344262 | 1.694915 |
| $\boldsymbol{\beta}$ | 0.5 | 0.55 | 0.45 | 0.02357 | 0.024720662 | 0.02236068 | 4.880884817 | -5.13167 |
| $\boldsymbol{r}$ | 0.5 | 0.55 | 0.45 | 0.02357 | 0.024152295 | 0.022973415 | 2.46950766 | -2.53206 |
| $\boldsymbol{\gamma}$ | 0.5 | 0.55 | 0.45 | 0.02357 | 0.024152295 | 0.022973415 | 2.46950766 | -2.53206 |
| N | 100 | 110 | 90 | 0.02357 | 0.022473329 | 0.0248452 | -4.653741075 | 5.409255 |
